# Single-embryo transfer reduces clinical pregnancy rates and live births in fresh IVF and Intracytoplasmic Sperm Injection (ICSI) cycles: a meta-analysis

**DOI:** 10.1186/1477-7827-7-36

**Published:** 2009-04-23

**Authors:** Ricardo LR Baruffi, Ana L Mauri, Claudia G Petersen, Andréia Nicoletti, Anagloria Pontes, João Batista A Oliveira, José G Franco

**Affiliations:** 1 Center for Human Reproduction – Prof Franco Junior, Ribeirão Preto, Sao Paolo, Brazil; 2 Department of Gynecology and Obstetrics, Botucatu Medical School, São Paulo State University – UNESP, Botucatu, Sao Paolo, Brazil

## Abstract

**Background:**

It has become an accepted procedure to transfer more than one embryo to the patient to achieve acceptable ongoing pregnancy rates. However, transfers of more than a single embryo increase the probability of establishing a multiple gestation. Single-embryo transfer can minimize twin pregnancies but may also lower live birth rates. This meta-analysis aimed to compare current data on single-embryo versus double-embryo transfer in fresh IVF/ICSI cycles with respect to implantation, ongoing pregnancy and live birth rates.

**Methods:**

Search strategies included on-line surveys of databases from 1995 to 2008. Data management and analysis were conducted using the Stats Direct statistical software. The fixed-effect model was used for odds ratio (OR). Fixed-effect effectiveness was evaluated by the Mantel Haenszel method. Seven trials fulfilled the inclusion criteria.

**Results:**

When pooling results under the fixed-effect model, the implantation rate was not significantly different between double-embryo transfer (34.5%) and single-embryo transfer group (34.7%) (*P *= 0.96; OR = 0.99, 95% CI 0.78, 1.25). On the other hand, double-embryo transfer produced a statistically significantly higher ongoing clinical pregnancy rate (44.5%) than single-embryo transfer (28.3%) (*P *< 0.0001; OR:2.06, 95% CI = 1.64,2.60). At the same time, pooling results presented a significantly higher live birth rate when double-embryo transfer (42.5%) (P < 0.001; OR: 1.87, 95% CI = 1.44,2.42) was compared with single-embryo transfer (28.4%).

**Conclusion:**

Meta-analysis with 95% confidence showed that, despite similar implantation rates, fresh double-embryo transfer had a 1.64 to 2.60 times greater ongoing pregnancy rate and 1.44 to 2.42 times greater live birth rate than single-embryo transfer in a population suitable for ART treatment.

## Background

Historically, embryos have been replaced in the uterus on either day 2 or 3 of development with resultant implantation rates of between 10% and 30% [1]. As a direct consequence of these low implantation rates, it has become an accepted procedure to transfer more than one embryo to the patient to achieve acceptable ongoing pregnancy rates. Thus, the transfer of more than a single embryo results in the finite probability of establishing a multiple gestation.

However, global efforts have been directed toward reduction of multiple gestations. Initiated by European researchers, this notion has resulted in the concept of elective single-embryo transfer (SET), which has achieved high popularity. Traditional arguments in favor of transferring a low number of embryos are based on the strongly augmented maternal and perinatal risks of multifetal pregnancy, increased social and political pressure and a decreased need for multifetal pregnancy reduction [2]. At the European Society of Human Reproduction and Embryology (ESHRE) consensus meeting in 2002, it was agreed that the preferred ART outcome should be the birth of one child and that a twin pregnancy should be considered a complication [3]. Nevertheless, the growth of SET is still very modest in Europe (12% in 2001 versus 13.5% in 2002, 15.7% in 2003 and 19.1% in 2004) [4]. Twin deliveries after IVF/ICSI are still close to 22% (2004) in Europe [4].

On the other hand, the Guidelines established by the ASRM and SART [5] recommend a limit of 1–2 cleavage-stage embryos for transfer even in favorable patients younger than 35 years. According to these Guidelines and depending on the woman's age and prognosis, the recommended number of embryos to transfer ranges between 2 and 5 (or even more "depending on individual circumstances after appropriate consultation"). Thus, in the USA the rate of SET is expressively lower than in Europe and presents low growth (6.2% in 2001 versus 6.7% in 2002, 7.5% in 2003, 8.2% in 2004 and 9.3% in 2005) [6]. In fact, shifting from high order embryo transfer (3 or more embryos) to double-embryo transfer (DET) apparently does not interfere in the results of IVF/ICSI cycles, while the option for SET instead of DET can lower pregnancy and live birth rates [7]. The present meta-analysis aims to compare current data on SET versus DET in fresh IVF/ICSI cycles with respect to implantation, ongoing clinical pregnancy and live birth rates.

## Methods

### Criteria for considering studies for this meta-analysis

All published and ongoing randomized controlled trials (RCT) compared current data on SET versus DET in fresh IVF/ICSI cycles

### Types of outcome measures

The outcome measures were implantation, ongoing clinical pregnancy and live birth rates.

### Identification of studies

Search strategies included online surveys of databases (MEDLINE, EMBASE, Science Citation Index, Cochrane Controlled Trials Register and OVID) from 1995 to 2008. There was no language restriction. The following headings and text strings were used: assisted reproductive technology, multiple pregnancy, randomized controlled trial, single-embryo transfer, and double-embryo transfer. The principal inclusion criterion was randomized controlled trial.

### Validity assessment and data extraction

Each trial was assessed independently by two reviewers and ranked for its methodological rigor and its potential to introduce bias. Missing data were obtained from the authors when possible.

### Statistical analysis

Data management and analysis were conducted using the StatsDirect statistical software (Cheshire, UK). The fixed-effect model was used for odds ratio (OR). Fixed-effect effectiveness was evaluated by the Mantel-Haenszel method. A confidence interval for the Mantel-Haenszel OR was calculated in the software StatsDirect using the Robins, Breslow and Greenland variance formula. A chi-square test statistic was used with its associated probability that the pooled OR was equal to 1. The measure of heterogeneity (non-combinability) was evaluated by Cochran's Q, the Breslow-Day and I^2 ^tests. A non-significant result (i.e. lack of heterogeneity) indicates that no trial has an OR that is significantly worse or better than the overall common OR obtained by pooling the data. Since a fixed-effect model has been employed herein, it is important to acknowledge that inferences refer only to the particular studies included in the analysis. Meta-analysis used in this manner is simply a device to pool the information from the various studies to provide a composite finding, but only for those studies.

## Results

### Search results

**Figure 1:**
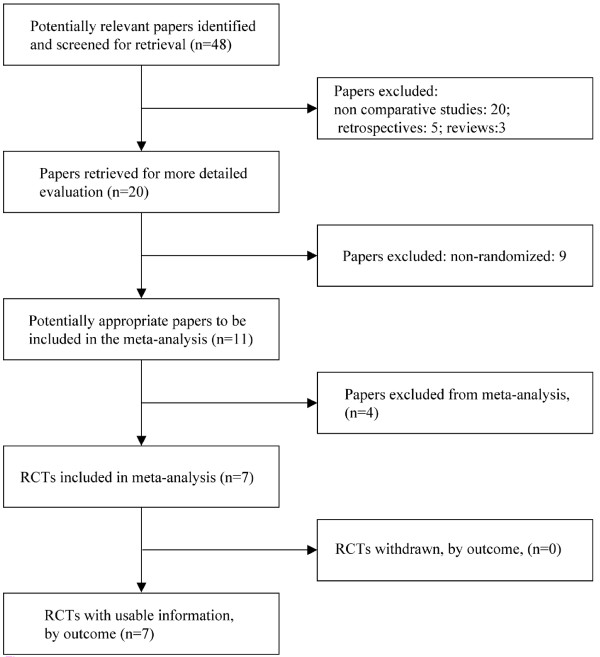
**QUOROM statement flow diagram illustrating selection of trials included in the meta-analysis**.

Seven trials fulfilled the inclusion criteria (Fig. 1).

### Description of the studies included

#### Gerris et al. [2]

This study aimed to obtain comparative data on the prospective implantation and pregnancy rates after SET and DET. First, top quality embryo characteristics were delineated by retrospectively analyzing embryos resulting in ongoing twin pregnancies after DET. A top quality embryo was characterized by the presence of 4 or 5 blastomeres at day 2 and at least 7 blastomeres on day 3 after insemination, the absence of multinucleated blastomeres and <20% cellular fragmentation on days 2 and 3 after fertilization. Using these criteria, a prospective study was conducted in women <34 years of age, who started their first IVF/ICSI cycle. In order to be included in the randomization, the patient had to agree to participate in the study and she had to have at least two top quality embryos at the time of embryo transfer. A total of 53 had produced at least two top quality embryos and were prospectively randomized. Randomization took place at the time of embryo transfer using external concealment. In all, 26 SET resulted in 17 conceptions (positive HCG), 14 clinical (53.8%) and 10 ongoing pregnancies (38.5%) with one monozygotic twin; 27 DET resulted in 22 conceptions; 21 clinical (77.8%) and 20 ongoing pregnancies (74%) with six sets of (30%) twins.

#### Martikainen et al. [8]

This multicenter study aimed to compare the effectiveness of SET and DET in a group of patients with good prognoses. An inclusion criterion in all four centers involved was at least four good quality embryos. A good quality embryo was defined as having symmetrical blastomeres and <20% fragmentation on day 2. A total of 144 couples agreed to participate in the study. They were randomized into the SET or DET groups by means of a computer-generated random number table. The laboratory personnel conducted the randomization just before embryo transfer (ET). The implantation rates of the fresh embryos transferred were quite similar between the SET and the DET groups (33.8% vs. 30.7%, respectively). The ongoing pregnancy rates were slightly but not significantly higher (29.7% vs. 40%) when two embryos were transferred.

#### Gardner et al. [9]

The objective of this study was to determine the efficacy of single blastocyst transfer. Participation in this study was offered to all patients undergoing IVF-ET with their own oocytes during a 24-month period who met the criteria for blastocyst stage ET. These criteria included a day-3 FSH = 10 mUI/ml, E2<80 pg/ml, normal endometrial cavity, and at least 10 follicles >12 mm in diameter on the day of HCG administration. Forty-eight patients were enrolled after informed consent was obtained. Patients were randomized at the time of transfer by a computer-generated table into either SET or DET transfer on day 5. SET resulted in an implantation and ongoing pregnancy rate of 60.9%, with no twins. DET resulted in an implantation rate of 56%, an ongoing pregnancy rate of 76% with a 47.4% incidence of twins.

#### Thurin et al. [10]

This study was designed to test the hypothesis that the rate of pregnancies resulting in at least one live birth in patients who had undergone the transfer of a single fresh embryo and, if no live birth resulted, the subsequent transfer of a frozen-and-thawed embryo, would be equivalent to the rate in patients submitted to the simultaneous transfer of two fresh embryos. Women were eligible for randomization if they were <36 years of age at the time of fresh ET, were undergoing their first or second *in vitro *fertilization cycle, and had at least two embryos of good quality available for transfer or freezing. Good-quality embryos included embryos with less than 20% fragmentation and 4 to 6 cells at day 2, 6 to 10 cells at day 3, or expanded blastocysts at day 5 or 6. An embryologist with the use of a computerized program performed randomization, at a ratio of 1:1, locally before the transfer, when the embryos could be evaluated. A total of 661 patients underwent randomization. Of those, 331 patients were randomly assigned to undergo DET and 330 to undergo SET. In fresh embryo transfer, the SET group had an ongoing pregnancy rate (28.4%) statistically significantly lower (*P *< 0.0001) than the DET group (44.1%). Also, the SET group had a live birth rate (27.6%) statistically significantly lower (*P *< 0.0001) than the DET group (42.9%)

#### Lukassen et al. [11]

Only patients undergoing their first IVF/ICSI cycle ever or the first cycle after a successful treatment were included. The age of the women had to be <35 years (at the time of ET) with a basal FSH level <10 IU/l. At least two embryos, with one excellent (grade 4) or one good (grade 3) quality embryo, had to be available for transfer on day 3 after oocyte retrieval: grade 4 = no blastomere fragmentation; grade 3=<10% fragmentation. A total of 107 patients were randomized into the SET (*n *= 54) or DET group (*n *= 53). The primary outcome, the ongoing pregnancy rate, was 25.9% in the SET group and 35.8% in the DET group.

#### Van Montfoort et al. [12]

This study performed a randomized controlled trial (RCT) to compare SET and DET in an unselected group of patients (i.e. irrespective of the woman's age or embryo quality). Patients who started their first IVF cycle were assessed for eligibility to participate in the study. All had to have normal fertilization of at least two oocytes (i.e. 2 PN embryos) in order to be randomly assigned to the SET or DET group. A total of 308 patients was included: 154 patients for SET and for 154 DET. Randomization was performed immediately prior to embryo transfer (using a non-transparent box containing sealed opaque envelopes). The clinical outcomes differed significantly between the SET and DET groups, with the respective percentages of positive pregnancy tests after transfer of fresh embryos being 33.1% versus 47.4%. The ongoing pregnancy rate after SET was significantly lower than in DET (21.4 vs. 40.3%, respectively) and the twin PR was reduced from 21.0% after DET to 0% after SET.

#### Moustafa et al. [13]

This study carried out a randomized controlled trial comparing SET versus DET in young women. Eighty-one patients undergoing embryo transfer in the assisted reproduction unit were prospectively included. Inclusion criteria were: women undergoing embryo transfer in a fresh cycle, at least one good quality embryo on the day of transfer, age ≤ 30 years at the time of embryo transfer, and no contraindication for pregnancy. Randomization was performed by a third party (a nurse) who was not involved in any other aspect of the study. The results showed that the live birth rate was not significantly different between SET (30%) versus DET (31.7%).

### Outcomes

#### Implantation rate (Fig 2)

**Figure 2:**
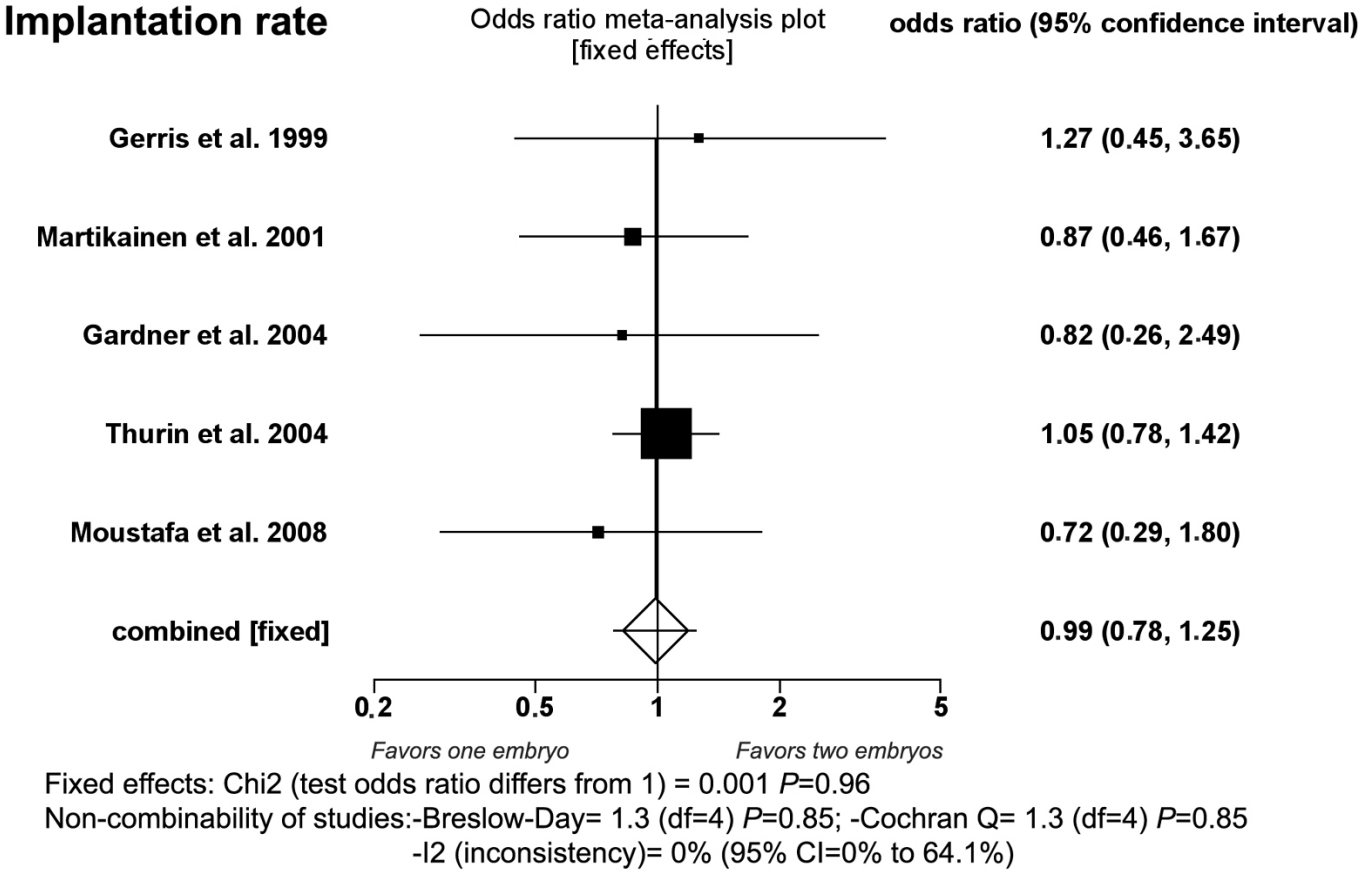
**Fixed-effect model**. Implantation rates after single- and double-embryo transfer.

Five studies were included [2,8-10,13]. The implantation rate was not significantly different between DET group (34.5%, 338/980) and SET group (34.7%, 163/470) (*P *= 0.96; OR = 0.99, 95% CI 0.78, 1.25). There was no heterogeneity in this comparison (Breslow-Day = 1.33, *df *= 4, *P *= 0.85; Cochran Q = 1.33, *df *= 4, *P *= 0.85; I^2 ^= 0%, 95% CI = 0% to 64.1%).

#### Ongoing clinical pregnancy rate (Fig. 3)

**Figure 3:**
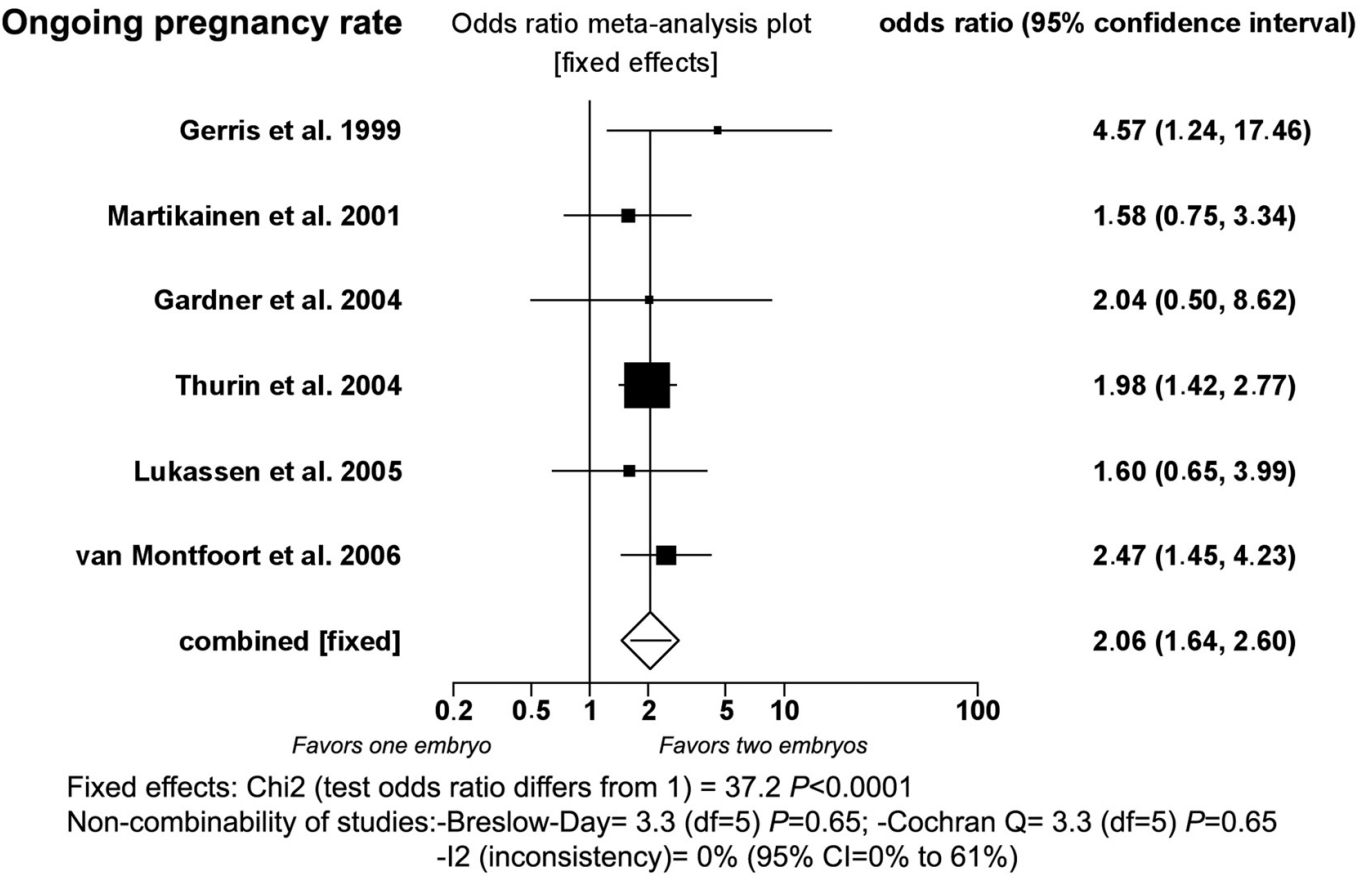
**Fixed-effect model**. Ongoing clinical pregnancy rates after single- and double-embryo transfer.

Six studies were included [2,8-12]. DET produced statistically significantly higher ongoing clinical pregnancy rate (44.5%; 294/660) than SET (28.3%; 187/661) when pooling results under the fixed-effect model (*P *< 0.0001; OR: 2.06, 95% CI = 1.64 to 2.60). There was no heterogeneity in this comparison (Breslow-Day = 3.31, df = 5, *P *= 0.65; Cochran Q = 3.3, df = 5, *P *= 0.65; I^2 ^= 0%, 95% CI = 0% to 61%).

#### Live birth rate (Fig. 4)

**Figure 4:**
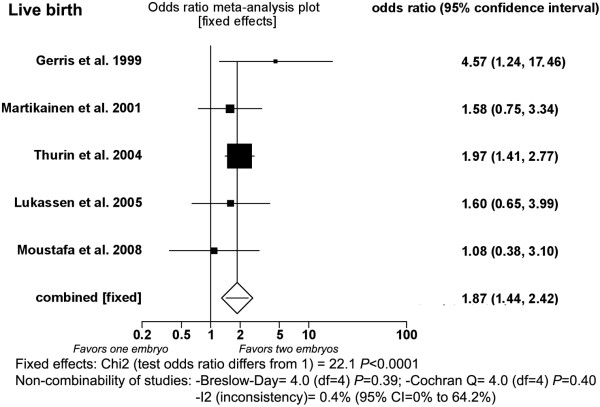
**Fixed-effect model**. Live birth rates after single- and double-embryo transfer.

Five studies were included [2,8,10,11,13]. Pooled results presented a significantly higher live birth rate when DET (42.5%; 222/522) (*P *< 0.001; OR: 1.87, 95% CI = 1.44,2.42) was compared with SET (28.4%; 149/524), a comparison without heterogeneity (Breslow-Day = 4.0, df = 4, *P *= 0.40; Cochran Q = 4.0, df = 4, *P *= 0.40; I^2 ^= 0.4%, 95% CI = 0% to 64.2%).

Meta-analysis with 95% confidence showed that fresh DET yielded a 1.64 to 2.60 times greater ongoing pregnancy rate and 1.44 to 2.42 times greater live birth rate that SET in a population suitable for ART treatment.

## Discussion

Although the risks should not be ignored, it appears excessive to condemn twin pregnancies as necessarily constituting an adverse result and thus to impose rigorous limits on DET utilization. In fact, the following factors that argue in favor of the DET option should be considered:

### Clinical pregnancy and live birth rates in a fresh IVF treatment cycle

SET in a fresh IVF treatment cycle reduces multiple births but also lowers live birth and pregnancy rates in comparison with double-embryo transfer. Van Montfoort et al. [12] demonstrated that applying SET in the first cycle of an unselected patient group would lead to a twin pregnancy rate of 0%. However, the price to be paid is a reduction of the ongoing pregnancy rate to approximately half of that obtained after DET. Roberts et al*. *[14], using a statistical model of live birth and twin outcomes in terms of routinely measured clinical parameters, demonstrated that the live birth rate would be reduced by up to 20–30% in order to achieve a 10% twin rate. Pandian et al. [7] showed that SET in a fresh IVF/ICSI treatment cycle reduces multiple births but also lowers pregnancy and live birth rates in comparison with DET. Our meta-analysis, which also showed statistically significantly higher ongoing clinical pregnancy and live birth rates in DET, updates the data with more recent studies and confirms this point.

Some authors [10,11,15] found that in a selected population of women, transferring one fresh embryo and then, if required, one or two frozen-thawed embryos significantly reduces the twin pregnancy rate without decreasing the overall pregnancy or live birth rate. However, the utility of such approaches depends crucially on embryo culture, selection and freezing policy [14]. Furthermore, this type of analysis, which compares the result of 2 transfers (fresh SET + frozen SET) with that of one transfer (fresh DET), can be considered biased. Cryopreservation cycles can be offered after DET as well, and the number of IVF/ICSI-SET cycles needed to obtain pregnancy rates comparable with DET cycles remains unknown [16]. On the other hand, if published studies and editorial opinions are scrutinized, their language usually refers to similar or acceptably high pregnancy rates in carefully selected patients, but never claims equivalency in pregnancy rates [17]

On the other hand, it was stated that we should be using the available data not to argue against SET, but rather, to find the optimal population of patients for whom it would be beneficial. Three of the studies of our meta-analysis included women >35 years old in the SET group, which may be considered inappropriate. However, when we carried out the analysis considering only the studies that included patients aged ≤ 35 years [2,10,11,13], the results were equal to those of meta-analysis considering all trials: Implantation rate: there was no significant difference between the DET group (33.8%, 267/790) and SET group (33.2%, 124/373) (*P *= 0.90; OR = 1.02 (95% CI = 0.78, 1.33); Ongoing pregnancy rate: DET produced statistically significantly higher ongoing clinical pregnancy rate (44.5%; 201/452) than SET (29.1%; 131/450) (*P *< 0.0001; OR:1.95, 95% CI = 1.48, 2.57) and Live birth rate: pooled results presented a significantly higher live birth rate when DET (42.9%; 194/452) (*P *< 0.0001; OR: 1.91, 95% CI = 1.45, 2.53) was compared with SET (28.2%; 127/450). Here also, as in the analysis considering all the studies, there was no heterogeneity in any comparison.

### Medical complications of a twin pregnancy

Twin pregnancies present substantial perinatal risks to both the mother and the infants. However, due to advances in health care, the maternal and perinatal risks have decreased. Nine out of 10 children born after 32 weeks of gestation usually present a good prognosis with a highly similar one-year survival rate between twins and singletons [18]. In addition, Pinborg et al. [19] showed that twins resultant from assisted reproduction present a risk of neurological sequelae similar to that of twins naturally conceived or even to singletons arising from assisted reproduction. Van Wely [16] affirms that although multiple gestations are associated with increased risk of complications for mothers and children, these complications occur in few twin gestations, with the majority resulting in the birth of two healthy babies. In addition, some authors [20,21] observed better results with twin pregnancies after assisted reproduction than those presented by spontaneously conceived twins.

On the other hand, Gleicher and Barad [22] reported that most risk assessments in the literature are calculated with pregnancy as the primary outcome, but in a fertility-treatment paradigm where patients want more than one child the statistically correct risk assessment should employ childbirth as the primary reference. Thus, two separate childbirths after SET (risks and complications) should be compared with one twin live birth. In this manner, these authors [22] showed that various twin pregnancy risks do not exceed the relative risk of 2.0, representing the combined risk of two singleton pregnancies required to achieve the same outcome as one twin delivery (two children). If we then further consider that IVF singleton pregnancies demonstrate higher adverse outcomes than spontaneously conceived singletons [20], it is possible to conclude that twin pregnancies (at least after IVF) do not represent higher overall outcome risks per newborn than singleton pregnancies [22].

### Embryonic synergism in sustaining implantation

Two studies have reported applying the concept of embryonic synergy by creating mathematical models that predict the implantation potential and compare the calculated results with the real results [23,24]. Both evidenced real multiple gestation (twin and triplets) rates higher than the mathematically calculated ones and suggested that this difference may reflect synergism of transferred embryos. Matorras et al*. *[24] estimated that the implantation probability is increased by 22% for each embryo additionally implanted. On the other hand, the lower risk of spontaneous loss in multiple gestations evidenced in some studies also corroborates the embryonic synergy concept. La Sala et al. [25] demonstrated that the rate of spontaneous fetal loss among singletons was significantly higher compared with losses among twins when only top-quality embryos had been transferred. Glujovsky et al*. *[26] showed that twin pregnancies have a lower chance of spontaneous embryo reduction than singletons. Lambers et al. [27] also report lower total pregnancy loss and loss per gestational sac in twin than in singleton gestations. Probably, the embryonic synergy, besides improving the local environment for implantation, also can promote a setting more favorable to pregnancy maintenance and embryonic growth [26]. However, the embryonic synergy concept has been disputed [14]. In this meta-analysis we did not observe any difference in the implantation rates or even survival rates (after 12 weeks) per embryo transfer for double- (29.9%) and single-embryo (29.2%) transfer (P = 0.78).

### Preference for a twin pregnancy in infertile patient

In the evaluation of twins, relevance applies not only to medical outcomes but also to the opinion of couples who have an unfulfilled wish for a child. For most couples who desire to conceive, the ideal number of children is two [28,29]. The largest preference study actually evaluated the preference for a twin or singleton pregnancy of mothers of IVF/ICSI twins (n = 266), IVF/ICSI singletons (n = 764) and mothers of spontaneous twins (n = 738) [30]. Mothers were approached with questionnaires at the time their children were 3–4 years of age and were asked whether they found either a singleton or twins more desirable as their first pregnancy. The study showed that 85% of IVF/ICSI-twin mothers and 62% of IVF/ICSI-singleton mothers would have preferred twins as their first delivery outcome compared with 60% of the non-IVF/ICSI-twin mothers. On the other hand, the couples apparently are more inclined to accept a twin pregnancy when they are not alerted to the risks [31]. However, this evidence is questionable. Murray et al*. *[32] showed that couples do not tend to take into account the risks associated with twin births even after counseling. Women embarking on IVF may be influenced more strongly by considerations of 'treatment success' rather than future risks to their offspring. Furthermore, their attitudes also seem to be more affected by regulations governing the number of attempts allowed, by the management of costs in the patients' country and by their knowledge of the current results with ART than by the possible risks [33].

**Figure 5:**
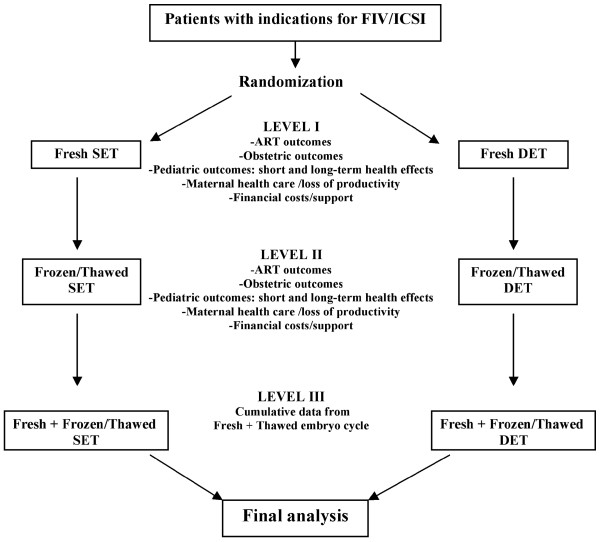
**Ideal flow chart for SET × DET comparison**.

In conclusion, DET poses diverse arguments in favor of its use whereas the indiscriminate application of SET appears unrealistic. SET represents an appropriate transfer option for only a small minority of IVF patients if pregnancy rates are to be satisfactory. The shift from DET to SET reduces the risk of twin pregnancy with prejudice that is important in pregnancy rates. Careful selection using couple characteristics and, crucially, embryo quality markers has the potential to mitigate the reduction in pregnancy rates in SET [14]. Further evaluations including an appropriate analysis of all short- and long-term results, all relevant costs and their funding sources are necessary for comparing the two embryo-transfer strategies (SET × DET). Figure 5 shows an ideal flow chart for SET versus DET comparison. It is only by a comprehensive approach that we will be able to decide whether SET or DET should be preferred.

## Competing interests

The authors declare that they have no competing interests.

## Authors' contributions

RB was responsible for designing and coordinating the study. All authors were responsible for data collection, data analysis, and data interpretation in the manuscript. RB, JO and JF were responsible for the statistical work and for writing the manuscript. JF was responsible for reviewing the manuscript. All authors read and approved the final manuscript.
